# Science or Tradition? Strength of Evidence for Footwear Fit Guidelines From Peer‐Reviewed Studies of People With Diabetes—A Foot in Diabetes UK Systematic Review

**DOI:** 10.1002/jfa2.70189

**Published:** 2026-07-20

**Authors:** Petra J. Jones, Christoforos Vlachopoulos, Helen Branthwaite, Krishna Gohil, Ryan Cassidy, Chris Morriss‐Roberts, Christian Pankhurst, Andrew Hill

**Affiliations:** ^1^ Leicester Diabetes Centre University Hospitals of Leicester Leicester General Hospital Leicester UK; ^2^ Diabetes Research Centre University of Leicester Leicester General Hospital Leicester UK; ^3^ Orthotics and Prosthetics Hampshire and Isle of Wight Healthcare NHS Trust UK; ^4^ Royal College of Podiatry Quartz House London UK; ^5^ Faculty of Health, Sport and Behavioural Sciences University of Northampton Northampton UK; ^6^ Buchanan Orthotics East Kilbride South Lanarkshire Glasgow UK; ^7^ London Metropolitan University London UK; ^8^ Orthotics Service Guy's and St. Thomas' NHS Foundation Trust Bowley Close Rehabilitation Centre London UK; ^9^ The SMAE Institute Maidenhead UK

**Keywords:** diabetes, evidence, fit, foot, footwear, shoes, ulcer

## Abstract

**Background:**

Footwear fit advice for patients and people with diabetes varies significantly. A review of the strength of evidence for footwear fit standards developed in peer‐reviewed studies is urgently needed both to inform future research and guidelines.

**Methods:**

We performed a systematic review to summarise and evaluate strength of evidence (GRADE) and risk of bias (Newcastle‐Ottawa Scale) of footwear fit standards in peer‐reviewed research studies involving people with diabetes through a search of Medline, Scopus, Embase and Web of Science databases.

**Results:**

Wearing incorrect shoe size increased DFU risk by up to 10 times (OR 1.7, *p* = 0.001 to OR 10.4, *p* < 0.001) based on 4 studies (risk of bias: poor (*n* = 3) to good (*n* = 1), but GRADE strength of evidence was Low to Moderate). GRADE strength of evidence for recommended footwear length, width and depth were very low to low. No studies were found examining DFU risk or in‐shoe pressure in shoelace alternatives, varying toe box shapes, heel width or height.

**Conclusion:**

Strength of evidence for current footwear fit guidelines is limited. Footwear fit guidance should currently be based on assessing adequate shoe size, with further research required to determine quantitative ranges for toe gaps, etc.

**Abbreviations**
CEGI: Internal footwear length gauge brand
CI: Confidence interval
cm: Centimetres
DFU: Diabetes‐related foot ulcers
FDUK: Foot in Diabetes UK
FIG: Footwear Interest Group comprised of FDUK, Royal College of Podiatry and other members
GRADE: Grading of Recommendations Assessment, Development and Evaluation
IWGDF: International Working Group on Diabetic Foot
kPa: Kilopascals, a unit of peak plantar pressure measurement
mm: Millimetres
N/cm^2^
: Newtons per square centimetre, a unit of peak plantar pressure measurement
NHS: National Health Service (United Kingdom healthcare provider)
NICE: National Institute for Health and Care Excellence
NOS: Newcastle Ottawa Scale Risk of bias
OR: Odds Ratio
PRISMA: Preferred Reporting Items for Systematic Reviews and Meta‐Analyses
PROSPERO: International Prospective Register of Systematic Reviews

## 1 Introduction

The prevalence of diabetes‐related foot ulcers (DFU) is increasing in many countries and continues to be a challenging global problem [1, 2, 3]. The average cost of treating a severe foot ulcer across seven World Health Organization regions was estimated as 17,000 dollars [4] and both the costs [5] and rate of recurrence [6] of DFU continues to be compared to some cancers. Mortality rates associated with DFU can be as high as 14% after only 1 year [7].

Footwear is often neglected in systematic reviews of risk factors related to either first‐ever diabetes‐related foot ulcers [8] or re‐ulceration [9]. When included, systematic reviews on DFU prevention focus only on therapeutic footwear that is pressure‐optimised [10] or custom‐made [11, 12, 13] or its design aspects (e.g., rocker bottom outsoles, etc.) [14]. However, non‐therapeutic footwear is often worn by the majority of people with diabetes—in some studies only 22%–35% regularly wear therapeutic footwear [15, 16] with even lower percentages (2.5%–5.3%) in some emerging and developing countries [17, 18].

NICE guidelines for diabetes foot recommend referring only those at moderate or high risk of foot ulcer to the foot protection service [19]. Even those at high risk are sometimes prescribed only up to two pairs of therapeutic footwear [20] and therefore utilise non‐therapeutic footwear for other needs (sports, social, religious, cultural or other contexts). It is known from a prior systematic review that wearing incorrectly fitting or inappropriate types of footwear can be related to diabetes foot ulcer causation [21]. However, the broader question of what strength of evidence exists within peer‐reviewed research studies for footwear fit recommendations more broadly is unanswered. An example of this is the considerable variation both in recommended minimum toe gaps (the difference between foot length and internal footwear length) from NHS healthcare providers which can be 0.6 cm (cm) [22], 1.0 cm [23] or even 1.5 cm [24] (Supporting Information S1: Table S1). This is confusing both for people with diabetes and the healthcare professionals wishing to advise them.

Our primary aim therefore was to undertake a systematic review to ascertain the strength of evidence for footwear fit recommendations within peer‐reviewed research studies involving people with diabetes. Footwear fit refers to the evidence for reported or recommended footwear length, width or depth. Secondary aims include evaluating the strength of evidence for specific toe box shapes, type of footwear fastening or heel heights, all of which may affect footwear fit and available space within footwear.

## 2 Materials and Methods

Foot in Diabetes UK (https://footindiabetes.org/) appointed a working group named the Footwear Interest Group (FIG) which comprised eight experts diabetes foot ulcer prevention through footwear to carry out this systematic review of the evidential basis for footwear fit standards within peer‐reviewed studies involving people with diabetes. This systematic review was performed using the Preferred Reporting Items for Systematic Reviews and Meta‐Analyses (PRISMA) guidelines [25] and both the protocol and search criteria were registered on the PROSPERO database (reference no. CRD420251069090).

### 2.1 Eligibility Criteria

Peer‐reviewed studies involving people with either type 1 or 2 diabetes mellitus were included which objectively measured or assessed footwear (direct physical examination of the feet and footwear) which specified quantifiable footwear fit standards (e.g., targets for adequate length, width or depth in footwear) or other outcomes of interest including toe box shapes, footwear fastening or heel height. Outcomes of interest included the utility of such standards in either reducing diabetes foot ulceration, lesions or in‐shoe pressure associated with footwear and footwear fit recommendations. Whilst evidence around recommendations related to wearing sandals as a footwear subtype were also gathered, these will be reported in a separate in‐depth paper on this topic that includes additional search criteria.

### 2.2 Data Sources and Search Criteria

**FIGURE 1:**
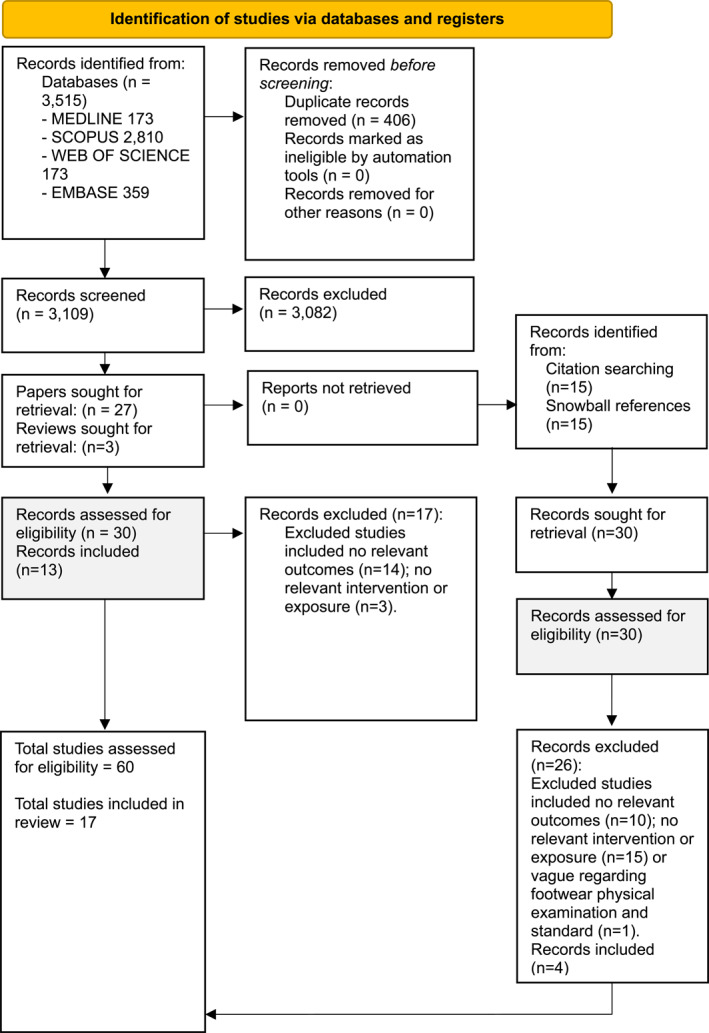
PRISMA diagram.

Medline, Scopus, Web of Science and EMBASE databases were searched until 21 July 2025 (Figure 1) for *peer‐reviewed studies* involving people with either type 1 or 2 diabetes mellitus which provided interventions for education about footwear; assessment of suitability of footwear; or diabetic foot ulcer prevention as described above (2.3–2.5) using the search criteria of (“diabetes” OR “diabetic”) AND (“footwear” OR “shoe” OR “shoes”) AND (“fit” OR “fitting” OR “size”). Data was extracted using a standardised spreadsheet template (authors, title, year, citation etc).

### 2.3 Study Selection

Two reviewers (CV and PJ) independently reviewed these papers to determine which of the 60 papers should be shortlisted, each blinded to each other's shortlist. Any discrepancies were resolved by a third reviewer (AH). This blinded process was then repeated to determine which studies should be included in the final review.

### 2.4 Risk of Bias and Quality of Evidence Assessments

Study risk of bias was independently assessed by two researchers (KG and CP) using the Newcastle‐Ottawa Scale blinded to each other's assessments [26]. Disagreements were resolved by a third reviewer (PJ). Grading of Recommendations for the Assessment, Development and Evaluation (GRADE) was used as a means of systematically rating strength of evidence. GRADE is a standardised framework used to rate the quality of scientific evidence within multiple studies to determine the strength of healthcare recommendations. GRADE provides a formal methodology for assessing representativeness and comparability of cohorts, ascertainment of exposure and adequacy of follow‐up for example. GRADE was utilised in this review to classify the evidence for each footwear fit recommendation (e.g., adequate footwear length targets) into one of four categories: High, Moderate, Low or Very low. GRADE was applied independently by two researchers (KG and CP) to evaluate the strength of evidence for each footwear fit recommendation area [26] for example recommended toe gap ranges with any disagreement resolved by the third reviewer (PJ).

## 3 Results

### 3.1 Search Results

Database searches yielded a total of 3515 records from which 406 duplicates were removed (Figure 1). From the remaining 3109 records, thirty papers were retrieved for full‐text examination (including 3 reviews). A further thirty papers were identified by citation searching (*n* = 15) and snowballing (*n* = 15) making sixty papers in total for full text examination. Thirty papers were shortlisted using the independent, blinded process described above from which 17 were included in the systematic review [27, 28, 29, 30, 31, 32, 33, 34, 35, 36, 37, 38, 39, 40, 41, 42, 43] (Figure 1). The remaining 43 studies were excluded given either no relevant outcomes (*n* = 24); no relevant intervention/exposure (*n* = 18); or unclear methodology (*n* = 1).

### 3.2 Incorrect Shoe Size and Risk of Foot Ulceration

Low to moderate strength of evidence (GRADE) was found that wearing incorrectly sized footwear increased DFU risk (OR 1.7, *p* = 0.001 [39] to OR 10.0, *p* = 0.016 [31], Table 1) based on four studies [31, 38, 39, 43] where risk of bias for each study ranged from poor [31, 38, 39] to good [43] (Newcastle‐Ottawa Scale: NOS). Some studies were assessed as poor given representativeness of the exposed/non‐exposed cohorts (e.g., elderly people on a rehabilitation ward with a low percentage of people with diabetes [31]) and/or adequacy of follow‐up period [31, 38, 39] (Supporting Information S1: Table S2).

Incorrectly sized footwear a UK half size longer than participant's feet increased risk of DFU by 10 times (*n* = 65, OR 10.0, *p* = 0.016) [31]. However, this study was based on older people (average age 82 years) of whom only 9% had diabetes (NOS rating: poor; Supporting Information S1: Table S2). Incorrectly sized footwear a shoe size larger or smaller than participant's feet worn by United States veterans (*n* = 440, 58% with diabetes) was associated with a five times higher risk of ulceration (OR 5.1, 95% CI 1.2–21.9, *p* = 0.02; NOS: poor) [38].

People with diabetes (*n* = 901) wearing either footwear one shoe size too large or small, slippers or footwear ‘with forced points’ were 10 times more likely to ulcerate (OR 10.4, 95% CI 4.5–24.1, *p* < 0.001) [43] with a low risk of bias (NOS: Good). Shoe with forced points on the feet were undefined but likely refer to signs of skin abrasion or impressions from footwear.

DFU causation was ascribed to footwear on the basis of clinical examination of feet/wounds and footwear fit [31, 38, 39, 43], combined with footwear condition [38], foot ulcer location and magnitude [39], or forced points on feet (presumably signs of footwear marking, rubbing or discolouring skin) [43].

### 3.3 Toe Gaps and Risk of Foot Ulceration

Toe gap ranges identified in previous narrative reviews [44, 45]: such as 1.0–2.0 cm [46] or 1.0–1.5 cm [47] were not linked to any evidence base (GRADE: very low to low, Table 2, Supporting Information S1: Tables S2 and S3). The only study found suggested that wearing ‘excessively long footwear’ (mean 0.9 cm toe gap ± 0.6 standard deviation vs. 0.5 ± 0.6 cm) was associated with callus formation in females with diabetes (*n* = 40) at the second metatarsal head (*p* = 0.026) [28]. However, this was not statistically significant for the entire cohort (*n* = 59).

**TABLE 1:** Risk of diabetes‐related foot ulceration (DFU)—relationship with wearing incorrect shoe sizes.

Lead author	Study size (*n*) [% DM]	Definition of incorrectly fitting footwear	Definition of inappropriate footwear	Strength of relationship with diabetes‐related foot ulcers and statistical significance	Newcastle ‐Ottawa scale Risk of bias
Burns [31]^a^	65 [9.0%]	Half shoe size [UK] larger or one width fitting larger	Fit only	**Incorrect footwear length & DFU: OR 10.0, *p* = 0.016** Incorrect footwear width & DFU: OR 0.75, *p* = 1.0	POOR
Nixon [38]	440 [58.4%]	1 shoe size larger/smaller [US]	Fit only	Size too big/small shoes & DFU: 93.3% people with DFU wearing poor fitting vs. 73.2% people without **OR 5.1 (95% CI 1.2‐21.9), *p* = 0.02**	POOR
Obimbo [39]	219 [100.0%]	1 shoe size smaller than feet [Kenya: UK equivalent]	Flip flops or sandals	Size too small or open footwear & DFU: 57.0% people with DFU wearing risky footwear (*p* = 0.001) 64.5% with complications vs. 23.6% without (95% DFU, 5% Charcot/cellulitis) **OR 1.73 (*p* = 0.001)**	POOR
Yazdanpanah [43]^a^	901 [100.0%]	1 shoe size larger/smaller [Iran: EU equivalent]	Slippers. shoes with forced points on the feet	Size too big/small footwear or slippers & DFU: 81.9% patients with DFU vs. 36.3% without **Adjusted OR 10.4 (95% CI 4.5‐24.1), p < 0.001**	GOOD

*Note:* Definition of incorrectly fitting footwear: UK = United Kingdom shoe sizes; US = United States shoe sizes. All statistically significant Odds Ratios are shown in bold.

Abbreviations: aOR, Adjusted Odds Ratio, CI, Confidence Interval; DFU, Diabetes‐related foot ulceration; OR, Odds Ratio.

^a^ Specific percentages for incorrectly fitting footwear in those with/without DFU unreported.

**TABLE 2:** GRADE certainty of evidence summary.

Outcome of interest	Certainty of the evidence (GRADE)	No. participants (no. Studies) [study types]	Comment
Shoe size fit	⊕⊕◯◯—⊕⊕⊕◯ LOW—MODERATE	7 studies (Table 1)	Four studies assessed shoe size in relation to ulcer risk [31, 38, 39, 43] (see Table 1 for details). A further three studies assessed toe gaps in relation to lesions/ulceration (Litzelman 1997 [33]), calluses (Amemiya 2020 [28]) and therapeutic footwear need (López‐Moral 2022) [34]
Standard measuring tools for fit	⊕◯◯◯ VERY LOW	0 studies (comparative)	No comparative studies evaluating use of alternative foot or footwear measuring tools. Evidence indirect from López‐Moral [34] (fit accuracy improved). Control group measuring tool not disclosed.
Footwear length	⊕◯◯◯‐⊕⊕◯◯ VERY LOW‐LOW	4 studies	Excess length of footwear identified as a risk factor for high external forces at MTH2 (Amemiya 2020 [28]) People with diabetes & neuropathy wore shorter shoes, direct ulcer outcome not powered (McInnes 2012 [35]). Incorrect British shoe size or half shoe size difference in length associated with self‐reported pain and ulceration within the elderly (Burns 2002 [31]). Insufficient length if toe gap < ¾ inches & too long if toe gap > ¾ inches. (Litzelman [ [33]). Largely subjective as a ‘nurse‐clinician's’ thumb used for this measurement. (See Supporting Information S1: Table S3 for details)
Footwear width	⊕◯◯◯‐⊕⊕◯◯ VERY LOW‐ LOW	3 studies	Observational evidence of pressure lesions in shoes narrower than feet; not diabetic‐specific nor adjusted for confounders. Incorrect footwear width & DFU: OR 0.75, *p* = 1.0 (Burns [31]) High pressure @ MTH5 attributable to tight footwear. β −0.305 (−2.021, −0.381) regression analysis (Amemiya [28]) Subjective width measurement OR 1.86, *p* = 0.07 correct width & DFU (Litzelman [33])^a^ (See Supporting Information S1: Table S5 for details)
Footwear depth	⊕◯◯◯ VERY LOW	0 studies	No study quantified toe‐box depth or vamp height in relation to ulcer risk; evidence inferred only from design guidelines.

Outcomes	Certainty of the evidence (GRADE)	No. participants (no. Studies) [study types]	Comment
Heel width	⊕◯◯◯ VERY LOW	0 studies	No empirical association studies found.
Heel height	⊕◯◯◯ VERY LOW	0 studies	No study examined heel elevation vs. plantar pressure or ulcer risk.
Toe box shapes	⊕◯◯◯ VERY LOW	0 studies	Evidence limited to guideline recommendations for rounded toe‐boxes; no measured risk ratios
Fastenings: Straps	⊕⊕⊕◯ MODERATE	1 study [case‐control]	Strap toe‐grip sandals caused ≈ 80% of footwear‐related first‐ever DFU; 20% first‐ever DFU caused by sandals without toe‐grips; adjustable fastenings protective [41]. No statistical significance reported.
Lace alternatives	⊕◯◯◯ VERY LOW	0 studies	No empirical association studies found.
Sandals/open footwear	⊕⊕⊕◯ MODERATE	5 studies (Table 3)	Strong observational link between open sandals and traumatic ulcers in neuropathic feet; consistent across African and Indian cohorts [32, 36, 39, 41, 42] (see Table 3 for details)

Grade	Definition
HIGH	We are very confident that the true effect is close to the estimate.
MODERATE	We have moderate confidence in the effect estimate. The true effect is likely close to the effect estimate, but there is a possibility that it is substantially different.
LOW	Our confidence in the estimate of the effect is limited: The true effect may be substantially different from the estimate.
VERY LOW	We have very little confidence in the estimate of the effect: The true effect is likely to be substantially different from the estimate.

^a^ Only Burns [31] included under width given dual use of shoe size and width fitting.

### 3.4 Footwear Width, DFU and Outcomes of Interest

Three objective quantitative standards were identified for footwear width which define incorrectly fitting footwear as (1) one shoe size [38, 39, 43] or (2) one width fitting larger or smaller than feet (4–7 mm) [31] or where (3) measured shoe width is not equal to foot width [28] (Supporting Information S1: Table S3). Strength of evidence was very low to low (GRADE) and limited to four studies [31, 38, 39, 43] (Table 2). Risk of bias scores (NOS) ranged from poor (*n* = 3 [31, 38], to good (*n* = 1, [43]). DFU outcomes from inadequate footwear width as distinct from inadequate length were examined in only one study where inadequate width (a half shoe size or one width fitting difference) was not statistically significant (OR 0.75, *p* = 1.0) [31].

Ulceration outcomes following evaluation of footwear width using tactile palpation [34, 36] were either not evaluated [34] or conflated with wearing inappropriate footwear [43]. Appropriate width footwear (based on subjectively assessed distance between eyestays) was found to increase the risk of non‐ulcerative lesions, cellulitis or ulcers (OR 3.1 (95% CI 1.1–9.2), *p* = 0.04) but the authors acknowledged subjectivity of assessment might be responsible [33].

No studies analysed in‐shoe pressures in relation to recommended width standards in footwear worn by people with diabetes. Only seven studies reported methodologies for measuring feet and footwear width which included callipers [31], slide rules [38, 39], measuring tape [42], 3D foot scanner [34], palpation [35] or eyestay visual inspection [33] (Supporting Information S1: Table S5).

### 3.5 Footwear Depth, Heel Width and Heel Heights

Only four of the 17 studies included in the review discussed footwear depth [27, 34, 42, 43] (Supporting Information S1: Table S6) which was subjectively described in all instances often with no methodology suggested for measurement [27, 42, 43]. 3D foot scanning feet and footwear palpation was the only suggested technique [33].

Only one of the seventeen studies specified maximum heel heights (2.0 cm [30]) but no evidence or reasoning for this threshold was provided and none of the studies analysed either in‐shoe pressures, risk of lesions or DFU in relation to heel heights (Supporting Information S1: Table S7). Adequate heel width to prevent footwear from slipping or rubbing was also neglected (Supporting Information S1: Table S8), (GRADE ratings: all very low).

### 3.6 Toe Box Shapes, Shoe Fastenings and Shoe Lace Alternatives

**FIGURE 2:**
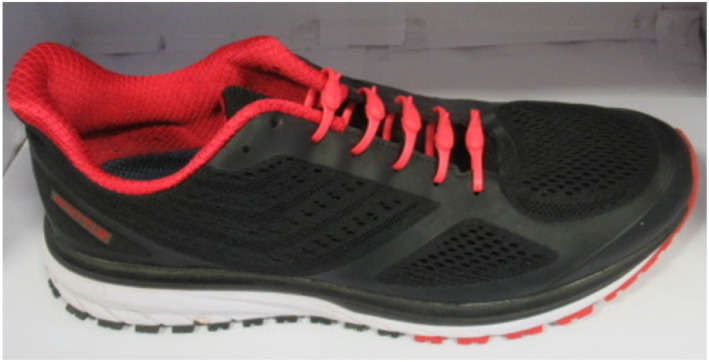
Shoelace alternatives—example of hickies.

None of the studies examined toe box shapes (Supporting Information S1: Table S9), or shoe lace shoe lace alternatives (Supporting Information S1: Table S10, Figure 2) in relation to in‐shoe pressures, risk of lesions or DFU (strength of evidence: very low).

## 4 Discussion

### 4.1 Previous Reviews of Footwear Fit

A systematic review by Buldt and Menz to determine the prevalence of incorrectly fitted footwear in studies that included, amongst other groups, people with diabetes found that 33%–82% of participants were wearing footwear with inadequate length, with only two studies reporting percentage wearing inadequate width [48]. However, strength of evidence for footwear fit standards was not explored. Two narrative reviews around footwear fit in diabetes foot ulcer prevention were carried out [44, 45] each based on a single database search (Google Scholar [44] and Medline [45]). Whilst these reviews revealed the lack of consensus on footwear fit (length [45], width and depth [44]), neither was systematic nor searched multiple databases. A subsequent systematic review focused specifically on the evidence that incorrect fitting footwear is a causal factor in diabetes‐related foot ulceration [21] and identified three studies where a statistically significant relationship existed.

Our systematic review remit was broader in encompassing toe box shape, heel height and footwear fastening recommendations as well as footwear fit recommendations more broadly (i.e., correctly or incorrectly fitting). We identified four studies with a statistically significant relationship between DFU and footwear fit.

### 4.2 Review Strengths and Weaknesses

Strengths of this review include the authors' multi‐disciplinary skillsets (research, podiatry, orthotics) with representation from Foot in Diabetes UK, the Royal College of Podiatry and Leicester Diabetes Centre. Other strengths include the absence of any start date restriction, use of broad terms such as ‘footwear or shoes’ and ‘fit, fitting or size’, the review size (3.1k excluding duplicates) and blinded assessment of risk of bias and strength of evidence (NOS/GRADE). Limitations include the restriction to English language papers and use of broad terms which may have accidentally omitted studies by not using specific phrases such as “heel height” etc.

### 4.3 Toe Gap Evidence and Footwear Measurement

**FIGURE 3:**
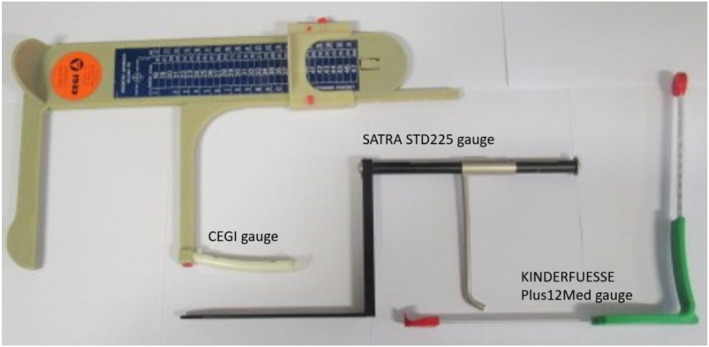
Footwear internal length measuring tools.

The review revealed an absence of evidence for toe gap ranges either in terms of DFU outcomes or in‐shoe pressures. During the shortlisting process for this review, three gauges were identified (Supporting Information S1: Table S4, Figure 3) for measuring internal footwear length (and thereby toe gap) [35, 49, 50] but not included within the review as DFU, wound, lesion or in‐shoe pressure outcomes were not assessed. The CEGI tool (pictured left, Figure 3) is the only gauge whose reliability has been formally assessed (Intraclass Correlation Coefficient 0.87 (95% CI: 0.80–0.92, *n* = 10) [49]. There is a need for research to formally assess both the accuracy and reliability of these quantitative methods used to measure internal footwear length when assessing adequacy of footwear length.

### 4.4 Footwear Width, In‐Shoe Pressure and Biomechanical Changes in Foot Width

In a study of people without diabetes (*n* = 10) wearing narrow rather than normal width fitting shoes, a 37 kPa (kPa) difference in in‐shoe pressures was found at the second to fourth metatarsal heads (270 kPa ± 52.3 vs. 232 kPa ± 70.4, *p* = 0.009 [51]). This suggests width fittings are likely to alter in‐shoe forefoot pressures similarly in those with diabetes but in our review very low to low strength of evidence was found. The recommendation that internal footwear width should equal width at the metatarsal phalangeal joints or the widest part of the foot assumes no significant change in orthogonal foot width occurs when walking rather than standing. A study of dynamic foot shape during walking in 19 people with diabetes suggested an additional 5 mm might be necessary for toe box designs to biomechanically accommodate changes in foot width at heel take‐off but lacked statistical significance [52].

### 4.5 Toe Box Shapes, In‐Shoe Pressures and Risk of Lower Extremity Amputation

The impact of toe box shapes upon either in‐shoe pressures, risk of lesions or DFU in people with diabetes also requires further research. ‘Fashion footwear’ (high‐heel pointed toe box shoes worn by women and narrow‐toed hard leather shoes worn by both sexes) worn once weekly was found to quadruple the risk of lower extremity amputation in people with diabetes (*n* = 223, OR 4.1, 95% CI 1.9–8.7, *p* < 0.001) [53]. Similarly, rounded toe box shapes significantly affected dorsal in‐shoe pressures at the hallux during walking in 27 female volunteers: rounded toe box: 16.7 ± 0.2 N/cm^2^; pointed toe box: 48.0 ± 0.5 N/cm^2^ [54].

### 4.6 Heel Heights and Footwear Fastenings

An evidence‐based maximum heel height to reduce average in‐shoe pressures below a clinically meaningful threshold, for example 200 kPa [55], is unfortunately still missing. Studies of healthy volunteers without diabetes suggest high heels require greater lower limb muscle effort [56] with a maximum heel height of 4 cm suggested to avoid impaired mobility or muscle imbalance [57].

An elastic shoelace alternative was found to have higher in‐shoe pressures during running (*n* = 11) [58] but such studies are rare and the effect of shoelace alternatives upon in‐shoe pressures, lesion or DFU risk have yet to be investigated. Where reel clutch shoelace alternatives were assessed in footwear worn by people with diabetes, plantar thermal stress response was compared only with loose or tight‐laced footwear rather than optimal lacing [59].

## 5 Conclusions

Evidence for footwear fit advice for people with diabetes is very limited. Footwear fit advice should currently be based around shoe sizes given the low to moderate strength evidence that wearing incorrect shoe size can increase the risk of DFU. Insufficient evidence currently is available to support recommended toe gaps, width, depth, heel width or heel height. Further research is urgently needed in these areas to provide clarity for people with diabetes and healthcare professionals alike.

## Author Contributions

**Petra J. Jones:** conceptualization, data curation, formal analysis, writing – original draft, writing – review and editing. **Christoforos Vlachopoulos:** writing – review and editing. **Helen Branthwaite:** writing – review and editing. **Krishna Gohil:** formal analysis, writing – review and editing. **Ryan Cassidy:** writing – review and editing. **Chris Morriss‐Roberts:** writing – review and editing. **Christian Pankhurst:** formal analysis, writing – review and editing. **Andrew Hill:** formal analysis, writing – review and editing.

## Conflicts of Interest

P.J. has received a grant (£15k) from Diabetes UK to assess footwear fit guidelines (toe gap ranges) under pressure in at‐risk feet (in‐shoe pressure and plantar thermal stress response, ClinicalTrials ID NCT06025422). P.J. attended a brainstorming workshop on 11/03/2025 with Renfrew Ltd, an engineering company based in Leicester to discuss ideas for devices to measure internal footwear width funded by a small grant (£4k) from the Institute of Precision Medicine, University of Leicester.

## Supporting information


Supporting Information S1

## Data Availability

The data that support the findings of this study are available from the corresponding author upon reasonable request.
