# Supplementary material for: Science or Tradition? Strength of Evidence for Footwear Fit Guidelines From Peer‐Reviewed Studies of People With Diabetes—A Foot in Diabetes UK Systematic Review
Source: J Foot Ankle Res. 2026 Jul 20;19(3):e70189. doi: 10.1002/jfa2.70189 (PMC13385204; doi:10.1002/jfa2.70189)
Supplement: Supplementary file 1 — Supporting Information S1 [file JFA2-19-e70189-s001.docx]

**Supplemental information

Table S1 NHS quantitative toe gap recommendations for patients**

|  | **Toe gap (cm)** | | **Date** | | **Audience** |
| --- | --- | --- | --- | --- | --- |
| **Lead author** | **Min** | **Max** | **Updated** | **Review** |  |
| [Dorset County Hospital](https://dchft.nhs.uk/leaflets/general-shoe-advice/) | 0.6 | 1.0 | 11/2021 | 11/2024 | General |
| [Royal Wolverhampton NHS Trust](https://royalwolverhampton.nhs.uk/wp-content/uploads/2025/06/Guide_to_buying_footwear.pdf) | 0.6 | 1.0 | 2016 | 5/2027 | Orthotics |
| [Sheffield Teaching Hospitals](https://publicdocuments.sth.nhs.uk/pil3645.pdf) | 0.6 | 1.0 | 9/2025 | 9/2028 | Orthotics |
| [East London NHS Foundation Trust](https://www.elft.nhs.uk/sites/default/files/2025-09/NHS%20Footwear%20leaflet%20.pdf) | 1.0 | -- | -- | -- | Podiatry |
| [East Sussex Healthcare NHS Trust](https://www.esht.nhs.uk/wp-content/uploads/2022/10/1018.pdf) | 1.0 | -- | 9/2022 | 9/2025 | General |
| [Essex Partnership University NHS](https://www.eput.nhs.uk/wp-content/uploads/2025/05/general-foot-care-information.docx) | 1.0 | -- | 5/2025 | -- | General |
| [Herefordshire & Worcestershire](https://www.hacw.nhs.uk/footwear/) | 1.0 | -- | -- | -- | General |
| [North Cumbria Integrated Care](https://www.ncic.nhs.uk/patients-visitors/patient-information-leaflets/podiatry-footwear-hosiery) | 1.0 | -- | 6/2023 | -- | Podiatry |
| [Oxford Health NHS Foundation Trust](https://www.oxfordhealth.nhs.uk/wp-content/uploads/sites/47/2024/02/Podiatry-Footwear-advice.pdf) | 1.0 | -- | 3/2024 | -- | Podiatry |
| [Somerset NHS Foundation Trust](https://www.somersetft.nhs.uk/podiatry/wp-content/uploads/sites/135/2022/04/Podiatry-Footwear-advice-FINAL-090322.pdf) | 1.0 | -- | 3/2022 | 3/2025 | Podiatry |
| [Suffolk GP Federation](https://suffolkfed.org.uk/wp-content/uploads/2025/07/Footwear-Advice-F213.pdf) | 1.0 | -- | 7/2025 | -- | Podiatry |
| [Torbay and South Devon](https://www.torbayandsouthdevon.nhs.uk/uploads/22229.pdf) | 1.0 | -- | 12/2022 | 12/2024 | Podiatry |
| [Ayrshire & Arran](https://www.nhsaaa.net/musculoskeletal-msk-service-patient-portal/foot-and-ankle-msk-patient-portal/footwear-advice-msk-patient-portal/) | 1.0 | 1.5 | -- | -- | MSK |
| [Leeds Community Healthcare](https://leedscommunityhealthcare.nhs.uk/our-services-a-z/podiatry/general-advice-leaflets/) | 1.0- 1.25 | -- | -- | -- | Podiatry |
| [University Hospital Southampton](https://www.uhs.nhs.uk/Media/UHS-website-2019/Patientinformation/Diabetes/Diabetes-managing-your-foot-wound-1473-PIL.pdf) | 1.0-1.25 | -- | 11/2023 | 11/2026 | Diabetes |
| [East Lancashire Hospitals](https://elht.nhs.uk/services/orthotics/patient-information/diabetes-and-orthotics) | 1.2 | -- | -- | -- | Diabetes |
| [Northern Care Alliance](https://www.northerncarealliance.nhs.uk/application/files/9116/3162/6198/footwear-advice.pdf)* | 1.5 | 2.5 | -- | -- | General |

Search criteria used:
“NHS” AND “trust” AND (“shoes” OR “footwear”) AND (“toe” OR “foot”) AND (“diabetes” OR "diabetic") AND ("fitted" OR "fitting”)
"diabetes footwear" AND "NHS Trust"
"NHS" AND ("longer" OR "longest") AND "toe" AND ("cm" OR "mm" OR "millimet" OR "centimet")
*Newer version no longer specifies any quantitative toe gap. [Link](https://www.northerncarealliance.nhs.uk/patient-information/patient-leaflets/diabetes-service-guide-looking-after-your-feet?q=%2Fpatient-information%2Fpatient-leaflets%2Fdiabetes-service-guide-looking-after-your-feet)

**Table S2 Newcastle-Ottawa risk of bias in studies assessing footwear worn by people with diabetes at risk of ulceration**

|  | **Selection** | | | | **Comparability** | **Outcomes** | | |  |
| --- | --- | --- | --- | --- | --- | --- | --- | --- | --- |
| **Lead author** | **Representativeness of exposed cohort** | **Selection of non-exposed cohort** | **Ascertainment of exposure** | **Outcome of interest** | **Comparability of cohorts** | **Assessment of outcome** | **Long enough follow-up** | **Adeq. follow up** | **Quality** |
| Abbott [27] | * | * | * | * | ** | * | * | * | GOOD |
| Amemiya [28] | * |  | * |  |  | * | * |  | POOR |
| Apelqvist [29] | * | * | * | * | ** | * | * | * | GOOD |
| Barwick [30] | * |  | * | * | ** | * |  |  | POOR |
| Burns [31] |  |  | * |  | * | * |  |  | POOR |
| Edrees [32] | * |  | * |  | * | * |  |  | POOR |
| Litzelman [33] | * | * | * | * | ** | * | * |  | GOOD |
| López-Moral [34] | * | * | * | * | ** | * | * | * | GOOD |
| McInnes [35] | * | * |  |  | ** | * |  |  | POOR |
| Monteiro-Soares [36] | * |  | * | * | * | * | * |  | GOOD |
| Ndip [37] | * |  | * |  | * | * |  |  | POOR |
| Nixon [38] | * | * | * |  | * | * |  |  | POOR |
| Obimbo [39] | * |  | * |  | * | * |  |  | POOR |
| Örneholm [40] | * |  | * |  | * | * |  |  | POOR |
| Premkumar [41] | * | * | * | * | ** | * |  |  | POOR |
| Sundram [42] | * |  | * |  | ** | * |  |  | POOR |
| Yazdanpanah [43] | * | * | * | * | ** | * | * | * | GOOD |

**Quality scoring criteria**
Good quality: 3 or 4 stars in selection domain AND 1 or 2 stars in comparability domain AND 2 or 3 stars in outcome/exposure domain
Fair quality: 2 stars in selection domain AND 1 or 2 stars in comparability domain AND 2 or 3 stars in outcome/exposure domain
Poor quality: 0 or 1 star in selection domain OR 0 stars in comparability domain OR 0 or 1 stars in outcome/exposure domain

**Table S3 Footwear length standards in peer reviewed studies involving people with diabetes**

| **Lead author** | **Recommended length standard** | **Findings** |
| --- | --- | --- |
| Abbott [27] | None (footwear type rather than fit) | Not applicable to footwear length |
| Amemiya [28] | None | 0.9 ± 0.6 cm mean toe gaps in callus formation (2^nd^ metatarsal) group vs. 0.5 ± 0.6 in non-callus group within female participants p=0.026 (No OR data). Differences in entire cohort (male & female) not significant |
| Apelqvist [29] | None (ill fitting is undefined) | Tight ill fitting shoes or socks verified in 123 of 314 people with diabetes foot ulcers, no statistical significance or breakdown into ill fitting shoes, ill fitting socks or both. |
| Barwick [30] | No length standard reported despite citation of IWGDF guidelines | Inadequate footwear associated with female sex OR 2.7 (1.4-5.2), p<0.05, critical peripheral artery disease OR 2.5 (1.1-5.9), and amputation OR 0.3 (0.1-0.7) p<0.05. |
| Burns [31] | Incorrect British shoe size or half shoe size difference of feet from footwear | Incorrect British shoe size or half shoe size difference in length associated ulceration in the elderly (OR 10.04, p=0.016) and self-reported pain in the elderly (p=0.0238) |
| Edrees [32] | None (footwear type rather than fit) | Not applicable to footwear length |
| Litzelman [33] | Less than a thumb’s width: ¾ inch (1.9cm) = too short More than a thumb’s width: ¾ inch (1.9cm) = too long | Insufficient length if toe gap < ¾ inches & too long if toe gap > ¾ inches. OR 1.84, p=0.07 correct shoe length indicative of wounds at follow-up |
| López-Moral [34] | 1.0cm toe gap. 1.5cm as threshold for excessive footwear length | Therapeutic footwear provided to four patients due to excessive footwear length and three patients due to toe compromise. |
| McInnes [35] | 1.0-1.5cm toe gap | 82% of participants with diabetes wore incorrect footwear length vs. 68% in controls without diabetes (p = 0.001).  35% wore footwear that was too short, 47% too long. |
| Monteiro-Soares [36] | Portuguese shoe size | Footwear classified as high risk if shoe size too small, low risk if correct size, presumably moderate risk if too big.  78% people with DFU wearing moderate-risk or high-risk footwear (p<0.001) |
| Ndip [37] | Subjective (“not too tight”) and no vents or buckles | 22% wore ill fitting footwear but unclear if this relates to entire cross-sectional cohort (n=300) or participants with DFU (n=39). No statistical significance. |
| Nixon [38] | 1 shoe size [US] larger/smaller | 93.3% people with DFU wearing poor fitting footwear vs 73.2% people without  OR 5.1 (95% CI 1.2-21.9), p=0.02 |

| **Lead author** | **Recommended length standard** | **Findings** |
| --- | --- | --- |
| Obimbo [39] | 1 shoe size [Kenya] smaller | Risky footwear & complications (including flip flops or sandals)  (95% DFU, 5% Charcot or cellulitis):  52.0% people with DFU wearing risky footwear vs  15.5% people without  OR 1.73 (p=0.001 |
| Örneholm [40] | None (ill fitting footwear is undefined) | 15% of footwear was ill fitting, p=0.05 |
| Premkumar [41] | None (footwear type rather than fit) | Not applicable to footwear length |
| Sundram [42] | None (footwear type rather than fit) | Not applicable to footwear length |
| Yazdanpanah [43] | 1 shoe size [Iran] larger/smaller | Ill-fitting footwear & DFU (including slippers or shoes with forced points):  OR 10.4 (95% CI 4.5-24.1), p<0.001 |

**Table S4 Shortlisted and included peer-reviewed study reported target footwear length for footwear worn by people with diabetes**

|  | **Toe gap** | |  |  | **Measurement method** | | **Toe gap source** |
| --- | --- | --- | --- | --- | --- | --- | --- |
| **Lead author** | **Min** | **Max** | **Size** | **%DM** | **Foot** | **Footwear** |  |
| Nancarrow [R1] | 1.0 | -- | 100 | 100.0 | -- | -- | -- |
| Tagang [R2] | 1.0 | -- | 156 | 100.0 | N/A | N/A | ADFN [G1] |
| Sundram [R3] | 1.0 | -- | 174 | 100.0 | Measuring tape | | Nancarrow |
| Chantelau [R4] | 1.0 | 1.5 | 568 100 | 100.0 0.0 | WMS | N/A | WMS [G2] |
| Chicharro-Luna [R5] | 1.0 | 1.5 | 108 | 100.0 | CEGI Pedometer | | Article [G3] |
| McInnes [R6] | 1.0 | 1.5 | 85 118 | 100.0 0.0 | Brannock | SATRA gauge | Chantelau study |
| Barwick [R7] | 1.0 | 2.0 | 726 | 23.6 | N/A | N/A | IWGDF [G4] |
| Isip [R8] | 1.0 | 2.0 | 170 | 100.0 | Brannock | Brannock | IWGDF |
| Qin [R9] | 1.0 | 2.0 | 1,007 | 100.0 | -- | -- | IWGDF |
| Fan [R10] | 1.25 | -- | 56 | 100.0 | -- | -- | RNAO [G5] |
| Reveal [R11] | 1.25 | 1.9 | 100 | 100.0 | Foot trace | Shoe trace | -- |
| López-Moral [R12] | 1.5 | -- | 30 | 100.0 | 3D scan | Palpation | -- |
| Meijer [R13] | 1.5 | 2.0 | 50 | 100.0 | -- | -- | Article [G6] |
| Tsuruoka [R14] | 1.6 | -- | 30 30 | 100.0 0.0 | -- | -- | -- |
| Lee [R15] | 2.0 | 3.0 | 165 | 100.0 | Brannock | Brannock | -- |

**KEY**
Toe gap sources:
Guidelines include ADFN: Australian Diabetes Foot Network Practical guideline;
IWGDF: International Working Group on Diabetic Foot; RNAO: Registered Nurses' Association of Ontario Best Practice Guidelines.
*Sourced via

**Table S4 References**

[R1] Nancarrow SA, Footwear suitability scale: A measure of shoe fit for people with diabetes. AJPM 1999; 33(2):57–62.
[R2] Tagang JI, Pei E, Chen R, Higgett N, Dahiru IL, Abdulrasheed I, Perceived role of therapeutic footwear in the prevention of diabetic foot ulcers: A survey of patients with diabetes mellitus in Kaduna State. Niger J Basic Clin Sci 2016; 13:78–84. https://doi.org/10.1016/j.dsx.2018.12.011
[R3] Sundram ER, Sidek MY, Yew TS, Types and grades of footwear and factors associated with poor footwear choice among diabetic patients in USM hospital. Int. J. Pub. Health & Clin. Sci. 2018; 5(2):2289–7577.
[R4] Chantelau E, Gede A. Foot dimensions of elderly people with and without diabetes mellitus – a data basis for shoe design. Gerontology. 2002; 48(4):241–4. https://doi.org/10.1159/000058357
[R5] Chicharro-Luna E, Ortega-Avila AB, Requena-Martínez A, Gijon-Nogueron G, Fit for purpose? Footwear for patients with and without diabetic peripheral neuropathy: A cross-sectional study. Primary Care Diabetes 2021; 15(1):145–149. https://doi.org/10.1016/j.pcd.2020.08.009
[R6] McInnes AD, Hasmi F, Farndon LJ, Church A, Haley M, Sanger DM, Vernon W. Comparison of shoe-length fit between people with and without diabetic peripheral neuropathy: A case- control study. Journal of Foot and Ankle Research 2012; 5: 9–17. https://doi.org/10.1186/1757-1146-5-9
[R7] Barwick AL, van Netten JJ, Reed LF, Lazzarini PA, Independent factors associated with wearing different types of outdoor footwear in a representative inpatient population: a cross-sectional study. JFAR 11, 19. https://doi.org/10.1186/s13047-018-0260-7
[R8] Isip JDJ, de Guzman M, Ebison A, Narvacan-Montano C. Footwear appropriateness, preferences and foot ulcer risk among adult diabetics at Makati Medical Centre Outpatient Department. J ASEAN Federation of Endocrine Societies JAFES. 2016;31(1):37–43 https://asean-endocrinejournal.org/index.php/JAFES/article/view/300
[R9] Qin Q, Oe M, Ohashi Y, Shimojima Y, Imafuku M, Dai M et al., Factors associated with the local increase of skin temperature, ‘hotspot’, of callus in diabetic foot: A cross-sectional study. JDST 2022; 16(5):1174-1182. https://doi.org/10.1177/19322968211011181
[R10] Fan L, Sidani S, Cooper-Brathwaite A, Metcalfe K. Effects of a foot self-care educational intervention on improving footwear choices in those with type 2 diabetes at low risk of foot ulceration. Diabetic Foot Canada. 2014;2:4–12. https://doi.org/10.1016/J.JCJD.2014.07.226
[R11] Reveal GT, Laughlin RT, Capecci P, Reeve FM, Foot and ankle survey in adults with diabetes mellitus. Foot & Ankle Int. 2001; 22(9):739–743. https://doi.org/10.1177/107110070102200910
[R12] López-Moral M, Clinical efficacy of a 3D foot scanner app for the fitting of therapeutic footwear in persons with diabetes in remission: A randomized and controlled clinical trial. Int. J. Lower Extremity Wounds 2022; 24(4): 937–944. https://doi.org/10.1177/15347346221124645
[R13] Meijer JWG, Links TP, Smit AJ, Groothoff JW, Eisma WH, Evaluation of a screening programme for diabetic foot complications. Prosthetics and Orthotics International 2001; 25:132–138. https://doi.org/10.1080/03093640108726586
[R14] Tsuruoka K, Oe M, Minematsu T, Tomida S, Ohashi Y, Shimojima Y, Mori Y, Nitta S et al., Skin characteristics associated with foot callus in people with diabetes: A cross-sectional study focused on desmocollin1 in corneocytes. J. Tissue Viability 2020; 29:291–296. https://doi.org/10.1016/j.jtv.2020.05.003
[R15] Lee WC, Foot and shoe survey in adult patients with diabetes mellitus. Korean Foot Ankle Soc. 2004; 8(2):153–156.
[G1] Bergin SM, Nube VL, Alford JB, Allard BP, Gurr JM, Holland EL, et al. Australian Diabetes Foot Network: Practical guideline on the provision of footwear for people with diabetes. J Foot Ankle Res. 2013; 6:6. https://doi.org/10.1186/1757-1146-6-6
[G2] WMS: Das Mass-System für Kinderschuhe. Richtlinien des ‘Arbeitkreises Kinderschuhe’ vom Dezember 1990. Offenbach/Main, Hauptverband der Deutschen Schuhindustrie e.V., 1990.
[G3] Edelstein JE, If the shoe fits: footwear considerations for the elderly, Phys. Occup. Ther. Geriatr. 1987; 5(4):1–16, http://dx.doi.org/10.1080/J148V05N0401
[G4] Bus S, Armstrong DG, Deursen R, Lewis J, Caravaggi C, Cavanagh P. IWGDF guidance on footwear and offloading interventions to prevent and heal foot ulcers in patients with diabetes. Diabetes Metab Res Rev. 2016; 32(S1):25:36. https://doi.org/10.1002/dmrr.2697

**Table S5 Footwear width standards in peer reviewed studies involving people with diabetes**

|  | **Width** | **Measurement methodology** | | **Source of recommendation** |
| --- | --- | --- | --- | --- |
| **Lead author** | **Recommendation** | **Foot** | **Shoe** |  |
| Abbott [27] | -- | -- | -- | -- |
| Amemiya [28] | “Tight width footwear” – no definition but reported -0.3cm average width gap in footwear | -- | -- | -- |
| Apelqvist [29] | “Ill fitting” is not defined (subjective) | -- | -- | -- |
| Barwick [30] | “Wide toebox” undefined (subjective) but IWGDF cited | -- | -- | IWGDF [46] |
| Burns [31] | Correct width fitting (incorrect if >1 width fitting difference) | Callipers | Callipers | --- |
| Edrees [32] | -- | -- | -- | -- |
| Litzelman [33] | “laced shoes, shoes were ‘too wide’ if the sides touched when laced and ‘too narrow’ if the sides were more than a  fingers width apart when laced” | Subjective | Subjective | Utah Department of Health Diabetes Control Program* |
| López-Moral [34] | “enough width to prevent scars from the presence of edema and sagittal deformities” | 3D foot scan | Palpation | Article: Bus (State of the art protocol)** |
| McInnes [35] | -- | -- | -- | -- |
| Monteiro-Soares [36] | -- | Palpation | Palpation | -- |
| Ndip [37] | -- | -- | -- | -- |
| Nixon [38] | One full size too large or small | Ritz stick | Ritz stick | -- |
| Obimbo [39] | “incorrect fitting one as a smaller shoe size than the foot” | Ritz stick | Ritz stick | -- |
| Örneholm [40] | -- | -- | -- | -- |
| Premkumar [41] | -- | -- | -- | -- |
| Sundram [42] | -- | -- | Measuring tape | Nancarrow [50]: Footwear suitability scale for diabetics |
| Yazdanpanah [43] | Width according to foot size | -- | -- | -- |

*No citation provided in Litzelman et al. [33]. IWGDF = International Working Group on Diabetic Foot
**Bus SA et al., State of the art design protocol for custom-made footwear for people with diabetes and peripheral neuropathy. Diabetes Metab Res Rev. 2019;e3237. https://doi.org/10.1002/dmrr.3237

**Table S6 Footwear depth standards in peer reviewed studies involving people with diabetes**

|  | **Depth** | **Measurement methodology** | | **Source of recommendation** |
| --- | --- | --- | --- | --- |
| **Lead author** | **Recommendation** | **Foot** | **Shoe** |  |
| Abbott [27] | Low risk footwear had ‘extra depth’ (subjective) | -- | -- | -- |
| Amemiya [28] | -- | -- | -- | -- |
| Apelqvist [29] | -- | -- | -- | -- |
| Barwick [30] | -- | -- | -- | -- |
| Burns [31] | -- | -- | -- | -- |
| Edrees [32] | -- | -- | -- | -- |
| Litzelman [33] | -- | -- | -- | -- |
| López-Moral [34] | “the box must be sufficiently high to accommodate foot deformities”* | 3D foot scan | Palpation | Article: Bus (State of the art protocol)** |
| McInnes [35] | -- | -- | -- | -- |
| Monteiro-Soares [36] | -- | -- | -- | -- |
| Ndip [37] | -- | -- | -- | -- |
| Nixon [38] | -- | -- | -- | -- |
| Obimbo [39] | -- | -- | -- | -- |
| Örneholm [40] | -- | -- | -- | -- |
| Premkumar [41] | -- | -- | -- | -- |
| Sundram [42] | (From Nancarrow: Sufficient depth to prevent deformity of the shoe by the digits) [50] | -- | -- | -- |
| Yazdanpanah [43] | Sufficiency of foot-wear height according to foot size | -- | -- | Review: Jones [44] |

**KEY**-- = Unreported. *Three patients were attributed as ill-fitting due to ‘compromise’ of the toe box but it’s unclear if this led to DFU.
**Bus SA et al., State of the art design protocol for custom-made footwear for people with diabetes and peripheral neuropathy. Diabetes Metab Res Rev. 2019;e3237. https://doi.org/10.1002/dmrr.3237

**Table S7 Heel height standards in peer reviewed studies involving people with diabetes**

|  | **Heel height (cm)** | | **Measurement method** | **Source of recommendation** |
| --- | --- | --- | --- | --- |
| **Lead author** | **Min** | **Max** |  |  |
| Abbott [27] | -- | “low” | -- | -- |
| Amemiya [28] | -- | -- | Tape measure | -- |
| Apelqvist [29] | -- | -- | -- | -- |
| Barwick [30] | -- | “low” (<2cm) | -- | Guideline: DFA^1^ Guideline: Bergin^2^ |
| Burns [31] | -- | -- | -- | -- |
| Edrees [32] | -- | -- | -- | -- |
| Litzelman [33] | -- | -- | -- | -- |
| López-Moral [34] | -- | -- | -- | -- |
| McInnes [35] | -- | -- | -- | -- |
| Monteiro-Soares [36] | -- | -- | -- | -- |
| Ndip [37] | -- | -- | -- | -- |
| Nixon [38] | -- | -- | -- | -- |
| Obimbo [39] | -- | -- | -- | -- |
| Örneholm [40] | -- | -- | -- | -- |
| Premkumar [41] | -- | -- | -- | -- |
| Sundram [42] | -- | -- | Tape measure | -- |
| Yazdanpanah [43] | -- | -- | -- | -- |

**KEY**
-- = Unreported. Heel height: cm = centimetres.
^1^ DFA = Diabetic Foot Australia guidelines: Van Netten JJ et al., Diabetic Foot Australia guideline on footwear for people with diabetes. J Foot Ankle Res. 2018; 11:2. doi: 10.1186/s13047-017-0244-z.
^2^ Bergin = Bergin SM et al. Australian Diabetes Foot Network: Practical guideline on the provision of footwear for people with diabetes. Journal of Foot and Ankle Research. 2013; 6(1):6. <https://doi.org/10.1186/1757-1146-6-6>. Source of 2 cm maximum heel height. No explanation for maximum heel height provided in the guideline.

**Table S8 Heel widths**

|  | **Heel width** | **Measurement methodology** | | **Source of recommendation** |
| --- | --- | --- | --- | --- |
| **Lead author** | **Recommendation** | **Foot** | **Shoe** |  |
| Abbott [27] | -- | -- | -- | -- |
| Amemiya [28] | -- | -- | -- | -- |
| Apelqvist [29] | -- | -- | -- | -- |
| Barwick [30] | -- | -- | -- | -- |
| Burns [31] | -- | -- | -- | -- |
| Edrees [32] | -- | -- | -- | -- |
| Litzelman [33] | -- | -- | -- | -- |
| López-Moral [34] | -- | -- | -- | -- |
| McInnes [35] | -- | -- | -- | -- |
| Monteiro-Soares [36] | -- | -- | -- | -- |
| Ndip [37] | -- | -- | -- | -- |
| Nixon [38] | -- | -- | -- | -- |
| Obimbo [39] | -- | -- | -- | -- |
| Örneholm [40] | -- | -- | -- | -- |
| Premkumar [41] | -- | -- | -- | -- |
| Sundram [42] | -- | -- | -- | -- |
| Yazdanpanah [43] | -- | -- | -- | -- |

**KEY**-- = Unreported i.e. no recommendations made within text of paper.

**Table S9 Toe box shapes**

|  | **Toe box** | **Source of recommendation** |
| --- | --- | --- |
| **Lead author** | **Recommendation** |  |
| Abbott [27] | -- | -- |
| Amemiya [28] | -- | -- |
| Apelqvist [29] | -- | -- |
| Barwick [30] | -- | -- |
| Burns [31] | -- | -- |
| Edrees [32] | -- | -- |
| Litzelman [33] | -- | -- |
| López-Moral [34] | -- | -- |
| McInnes [35] | -- | -- |
| Monteiro-Soares [36] | -- | -- |
| Ndip [37] | -- | -- |
| Nixon [38] | -- | -- |
| Obimbo [39] | -- | -- |
| Örneholm [40] | -- | -- |
| Premkumar [41] | -- | -- |
| Sundram [42] | -- | -- |
| Yazdanpanah [43] | -- | -- |

**KEY**-- = Unreported

**Table S10 Shoe fastening recommendations in peer reviewed studies involving people with diabetes**

|  | **Recommended shoe fastenings** | | | | | **Source of recommendation** |
| --- | --- | --- | --- | --- | --- | --- |
| **Lead author** | **Laces** | **Velcro** | **Strap** | **Buckle** | **Other** |  |
| Abbott [27] | YES | -- | NO | NO | -- | Buckles=mod. Risk^1^ Sandals = high risk |
| Amemiya [28] | -- | -- | -- | -- | -- | -- |
| Apelqvist [29] | -- | -- | -- | -- | -- | -- |
| Barwick [30] | YES | YES | -- | -- | -- | Guideline: Bergin^2^ |
| Burns [31] | -- | -- | -- | -- | -- | -- |
| Edrees [32] | -- | -- | -- | -- | -- | -- |
| Litzelman [33] | YES | -- | -- | -- | -- | -- |
| López-Moral [34] | YES | -- | -- | YES | -- | -- |
| McInnes [35] | -- | -- | -- | -- | -- | -- |
| Monteiro-Soares [36] | YES | -- | -- | -- | -- | -- |
| Ndip [37] | -- | -- | -- | NO | -- | -- |
| Nixon [38] | -- | -- | -- | -- | -- | -- |
| Obimbo [39] | -- | -- | -- | -- | -- | -- |
| Örneholm [40] | -- | -- | -- | -- | -- | -- |
| Premkumar [41] | -- | -- | -- | -- | -- | -- |
| Sundram [42] | YES | -- | YES* | -- | -- | -- |
| Yazdanpanah [43] | -- | -- | -- | -- | -- | -- |

**KEY**
-- = Unreported. * Adjustable straps
^1^ Buckle footwear fastenings are included in the classification of ‘moderate risk’ whereas sandals (presumably with straps) are classified as ‘high risk’.
^2^ Bergin = Bergin SM et al. Australian Diabetes Foot Network: Practical guideline on the provision of footwear for people with diabetes. Journal of Foot and Ankle Research. 2013; 6(1):6. https://doi.org/10.1186/1757-1146-6-6
